# Using an mHealth App (iGAM) to Reduce Gingivitis Remotely (Part 2): Prospective Observational Study

**DOI:** 10.2196/24955

**Published:** 2021-09-16

**Authors:** Guy Tobias, Assaf B Spanier

**Affiliations:** 1 Department of Community Dentistry Faculty of Dental Medicine The Hebrew University-Hadassah School of Dental Medicine Jerusalem Israel; 2 Department of Software Engineering Azrieli College of Engineering Jerusalem Israel

**Keywords:** mHealth, public health, oral health promotion, gum health, COVID-19

## Abstract

**Background:**

Gingivitis is a nonpainful, inflammatory condition that can be managed at home. Left untreated, gingivitis can lead to tooth loss. Periodic dental examinations are important for early diagnosis and treatment of gum diseases. To contain the spread of the coronavirus, governments, including in Israel, have restricted movements of their citizens which might have caused routine dental checkups to be postponed.

**Objective:**

This study aimed to examine the ability of a mobile health app, iGAM, to reduce gingivitis, and to determine the most effective interval between photograph submissions.

**Methods:**

A prospective observational cohort study with 160 unpaid participants divided into 2 equal groups using the iGAM app was performed. The intervention group photographed their gums weekly for 8 weeks. The wait-list control group photographed their gums at the time of recruitment and 8 weeks later. After photo submission, the participants received the same message “we recommended that you read the information in the app regarding oral hygiene habits.” A single-blinded researcher examined all the images and scored them according to the Modified Gingival Index (MGI).

**Results:**

The average age of the intervention group was 26.77 (SD 7.43) and 28.53 (SD 10.44) for the wait-list control group. Most participants were male (intervention group: 56/75,74.7%; wait-list control group: 34/51, 66.7%) and described themselves as “secular”; most were “single” non-smokers (intervention group: 56/75, 74.7%; wait-list control group: 40/51, 78.4%), and did not take medications (intervention group: 64/75, 85.3%; wait-list control group: 40/51, 78.4%). A total of 126 subjects completed the study. A statistically significant difference (*P*<.001) was found in the dependent variable (MGI). Improvements in gingival health were noted over time, and the average gingivitis scores were significantly lower in the intervention group (mean 1.16, SD 1.18) than in the wait-list control group (mean 2.16, SD 1.49) after 8 weeks. Those with more recent dental visits had a lower MGI (*P*=.04). No association was found between knowledge and behavior. Most participants were familiar with the recommendations for maintaining oral health, yet they only performed some of them.

**Conclusions:**

A dental selfie taken once a week using an mobile health app (iGAM) reduced the signs of gingivitis and promoted oral health. Selfies taken less frequently yielded poorer results. During the current pandemic, where social distancing recommendations may be causing people to avoid dental clinics, this app can remotely promote gum health.

## Introduction

### COVID-19 and Dental Implications

One and a half years have passed since the COVID-19 pandemic broke out in Wuhan, China [1]. On March 19, 2019, the Israeli Prime Minister declared a national state of emergency and various restrictions were enforced. “Normal” life resumed with ongoing rules of wearing masks, hand hygiene, and keeping 2 meters between people. Dentistry is a field with close contact between patients and the clinical team, with a high risk of transmission of infections [2]. The aim of these restrictions is to balance the public health issues, which include decreasing or preventing the spread of COVID-19, with the need for minimizing dental and oral pain during this period.

### Dental Background

The diseases of the oral cavity can be divided according to the type of affected tissue: soft or hard tissue disease. Bacteria in the dental plaque are the main cause of these diseases [3].

#### Hard Tissue Disease

Caries or tooth decay [4] affects over 90% of the world’s population. This is caused by bacterial plaque on tooth surfaces that convert the sugars from food into acids which then cause minerals to leech out of the outer layer of the tooth (enamel) and dissolve it. The bacteria then penetrate the deeper layers of the teeth, namely the dentine and eventually the pulp. Tooth decay, also known as caries, is a progressive disease that can be painful and cause systemic infection and thus cannot be ignored.

#### Soft Tissue Disease

Gingivitis [5] is a nonpainful, reversible inflammatory condition characterized by swelling and redness, along with occasional bleeding when brushing and bad breath. This reversible stage can be treated with home remedies including strict oral hygiene, routine dental visits, and cleanings by a dental hygienist. Untreated gingivitis can progress into periodontitis, where irreversible damage to the tooth-supporting tissues can lead to tooth mobility and tooth loss.

Periodic dental examinations are important for the early diagnosis and treatment of dental problems [6], and the issue of pain figures prominently in the decision to go to the dental clinic [7-9]. Between visits, dentists do not know what changes are occurring in their patients’ mouths and patients usually do not notice changes in the oral cavity until they experience pain. During this period of lockdown and avoidance of routine dental checkups, we speculate that many people are experiencing a decline in their oral health.

#### eHealth and mobile health

eHealth [10] is the use of information and communication technologies for health. Mobile health (mHealth) is a component of eHealth [11] and is defined by the Global Observatory for eHealth as “medical and public health practice supported by mobile devices, such as mobile phones, patient monitoring devices, personal digital assistants, and other wireless devices” [12].

There are currently over 318,000 mHealth apps, with hundreds of new apps added daily. mHealth apps can monitor health conditions and alert the patient or attending physician about deterioration [13,14].

In August 2020, we published a paper describing the development of an mHealth app in the field of dentistry named iGAM [15]. The paper presented here is the second part in the series and presents the results of an observational study conducted to examine the ability of a cellular app to reduce gingivitis. The third part will include the results of a mixed methods study that investigated the acceptance of this type of mHealth app. Using computerized technologies in dentistry can assist in remotely monitoring patients during times of social restrictions (eg, COVID-19). A study conducted in Italy by Giudice et al [16] in 2020 found that teledentistry minimizes contact and therefore decreases COVID-19 dissemination. Another study noted the urgent need to incorporate mHealth apps into routine dental practice as a complementary tool to manage with those patient needs that arise due to social distancing [17].

To the best of our knowledge, there are currently no mHealth apps that monitor gingival health. The aim of this observational study was to examine the ability of an mHealth app, iGAM, to reduce gingivitis without researcher intervention and to determine the best interval between photograph submissions.

## Methods

### Design and Development

This prospective observational study was conducted between September 2019 and May 2020 at the Department of Community Dentistry Faculty of Dental Medicine, The Hebrew University-Hadassah School of Dental Medicine. The protocol was approved by the Hadassah research ethics committee (IRB 0212-18-HMO), and written informed consent was obtained from all participants. There was no payment for participation. Advertisements were posted in academic institutions and hospitals in Jerusalem for 1 month (August 2019). Potential participants met with the primary investigator (GT), and, after a thorough explanation about the study, they could download the app and fill in the informed consent form. The first participant was randomly assigned by flipping a coin to either the intervention group or wait-list control group, and from that point onward, the app was assigned the groups such that sequential enrollees were assigned to different groups. The intervention group photographed their gums once a week for 8 weeks. The wait-list control group photographed their gums at the time of recruitment for the study and at the end of the study 2 months later. During the first login, the users set a password known only to them and were then asked to provide anonymous personal information. The data were then stored on a Google encrypted server, Firebase. Each participant was given a randomly generated user number.

This study used the Modified Gingival Index (MGI) [18] that was introduced in the mid-1980s and was found to be reliable in the visual diagnosis of gingivitis and for assessment of the prevalence and severity of gingivitis without the use of any dental instruments (ie, this index can be used in a home setting). This index focuses on the gingival unit, which includes 3 parts: (1) the free gingiva, the part of the gums around the tooth not attached to the tooth surface; (2) the attached gingiva, the firm part of the gums that is tightly attached to underlying tissues of the tooth; and (3) the alveolar mucosa, the soft tissue between the lips and the gums. The index has a scale from 0 to 4, which describes the relative status of the gums as follows: 0=absence of inflammation; 1=mild inflammation—slight change in color, and small area of altered texture but not the entire marginal or papillary gingival unit; 2=mild inflammation—as above but involving the entire marginal or papillary gingival unit; 3=moderate inflammation—glazing, redness, edema, and/or hypertrophy of the marginal gingival unit; and 4=severe inflammation—marked redness, edema and/or hypertrophy of the marginal or papillary gingival unit, spontaneous bleeding, congestion, or ulceration.

After registration, the user was transferred to the part of the app where the photos of the gums are taken. Upon submission of the photograph, the researcher (AS) received an SMS text message, “User (number) submitted a photograph.” The user received an SMS text message, “The photograph was submitted,” with the submission date and the number of remaining submissions required. Participants received a comprehensive dental kit: toothpaste, toothbrush, mouthwash, dental floss, and toothpicks. In addition, the participants received a reusable mouth opener. The participants were taught how to use the app and take photos of their own gums while using the mouth opener. In addition, participants were instructed to photograph their gums during the day in front of a mirror using white lighting with a white wall in the background. After each photo submission, the participants received the following message “we recommended that you read the information in the app regarding oral hygiene habits.”

**Figure 1:**
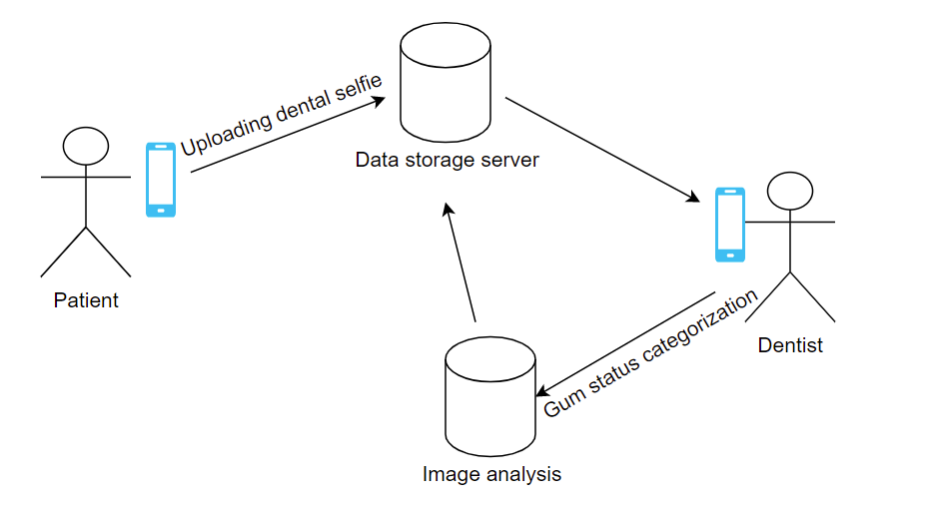
Interaction between participant (patient), app admin (researcher, dentist), and data storage server (Firebase).

All tutorials and instructions were performed by a single researcher (GT) who was blinded to the treatment group and who examined the images and scored them according to the MGI index; the results were not reported to the participants. Figure 1 summarizes the process.

Interactions with the participants were via text messages from within the app. There was only 1 training session, and participants who asked questions were referred to the training documents in the app.

### Inclusion Criteria

The inclusion criteria for participants included 3 points: (1) over the age of 18 years, (2) could read and understand Hebrew, and (3) in possession of a smartphone with an Android operating system

The iGAM app is currently available on the Google Play Store in Hebrew. The app’s development was described in a recently published article.

### iGAM features

The iGAM mHealth app has 3 components: (1) a self-completion questionnaire that deals with knowledge and attitudes toward oral hygiene habits; (2) text accompanied by illustrations regarding brushing techniques as well as short articles about the importance of maintaining oral health in general and during pregnancy, the implications of smoking on gum health, and the connection between gum diseases and general health; and (3) the ability to photograph one’s own gums using the rear camera of the smartphone. The first volunteer was allocated to the intervention group, the second volunteer to the wait-list control group, and so on.

### Statistical Methods

Baseline characteristics are presented as mean and SD for continuous variables and as frequencies and percentages for categorical variables. Specific statistical tests are detailed in the results section. The data were analyzed with SPSS statistics software version 27.0 (IBM Corporation), and significance levels were set at a *P* value of .05.

## Results

### Demographic and Descriptive Statistics

**Figure 2:**
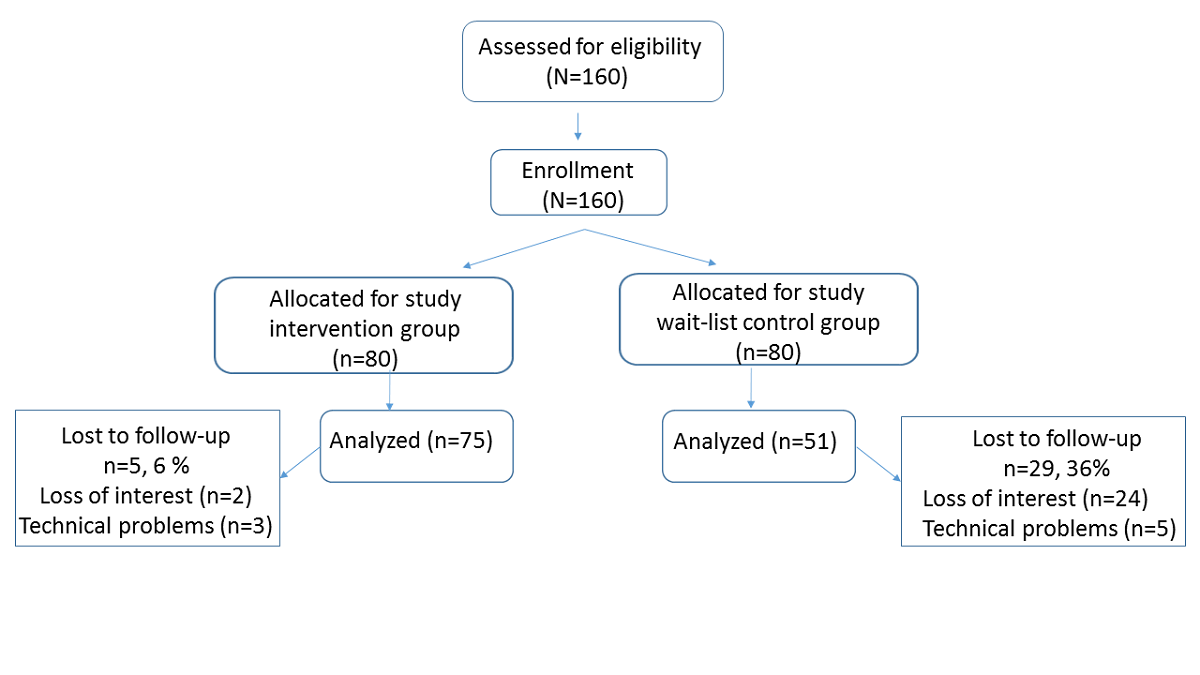
Distribution of study participants.

In total, 175 candidates responded to the recruitment advertisement. The study included 160 participants who met the inclusion criteria and were divided into 2 equal groups. Participants in the intervention group took weekly photographs of their gums for 8 weeks. Those in the wait-list control group were asked to photograph their gums at 2 time points: at the beginning of the study and 8 weeks later. In all, 126 participants successfully completed the tasks: 75 (59.5%) from the intervention group and 51 (40.5%) from the wait-list control group. Figure 2 shows the distribution of the study participants.

The age range of the intervention group was 19 to 65 years with an average of 26.77 (SD 7.43), while that in wait-list control group was from 20 to 66 years with an average of 28.53 (SD 10.44).

In the comparison of the intervention group and the wait-list control group, the majority were male (intervention group: 56/75, 74.7%; wait-list control group: 34/51, 66.7%), native to Israel (intervention group: 73/75, 97.3%; wait-list control group: 50/51, 98%), and Jewish (intervention group: 75/75, 100%; wait-list control group: 49/51, 96.1%); the common term regarding the level of religiosity in both groups was “secular” (intervention group: 21/75, 28%; wait-list control group: 15/51, 29.4%). In addition, the most common value in terms of marital status was “single” (intervention group: 38/75, 50.7%; wait-list control group: 24/51, 47.1%). The majority of the participants in the wait-list control group were employees (24/51, 47.1%), while in intervention group, the most common term from an occupational point of view was “student” (31/75, 41.3%). Most of the study population did not smoke (intervention group: 56/75, 74.7%; wait-list control group: 40/51, 78.4%) and did not take any medications (intervention group: 64/75, 85.3%; wait-list control group: 40/51, 78.4%; see Table 1).

**Table 1:** Characteristics of the study population.

Sociodemographic characteristics		Intervention group, n (%) (N=75)	Wait-list control group, n (%) (N=51)
**Gender**			
	Male	56 (75)	34 (67)
	Female	19 (25)	17 (33)
**Country of Birth**			
	Israel	73 (97)	50 (98)
	Other	2 (3)	1 (2)
**Nationality**			
	Jewish	75 (100)	49 (96)
	Other	0/0 (0)	2 (4)
**Religiosity**			
	Secular	21 (28)	15 (29)
	Traditional	14 (19)	10 (20)
	Orthodox	15 (20)	10 (20)
	Ultra-orthodox	17 (23)	7 (14)
	Refused to answer	8 (11)	9 (18)
**Family Status**			
	Married	27 (36)	14 (27)
	Living with a partner	7 (9)	6 (12)
	Divorced	2 (3)	5 (10)
	Widow	1 (1)	1 (2)
	Single	38 (51)	24 (47)
	Refused to answer	0/0 (0)	1 (2)
**Occupation**			
	Salaried employee	28 (37)	24 (47)
	Self-employed	13 (17)	7 (14)
	Unemployed	2 (3)	0 (0)
	Student	31 (41)	19 (37)
	Retiree	0 (0)	1 (2)
	Soldier	1 (1)	0 (0)
**Smoker**			
	Yes	19 (25)	11 (22)
	No	56 (75)	40 (78)
**Taking medication**			
	Yes	11 (15)	11 (22)
	No	64 (85)	40 (78)

**Figure 3:**
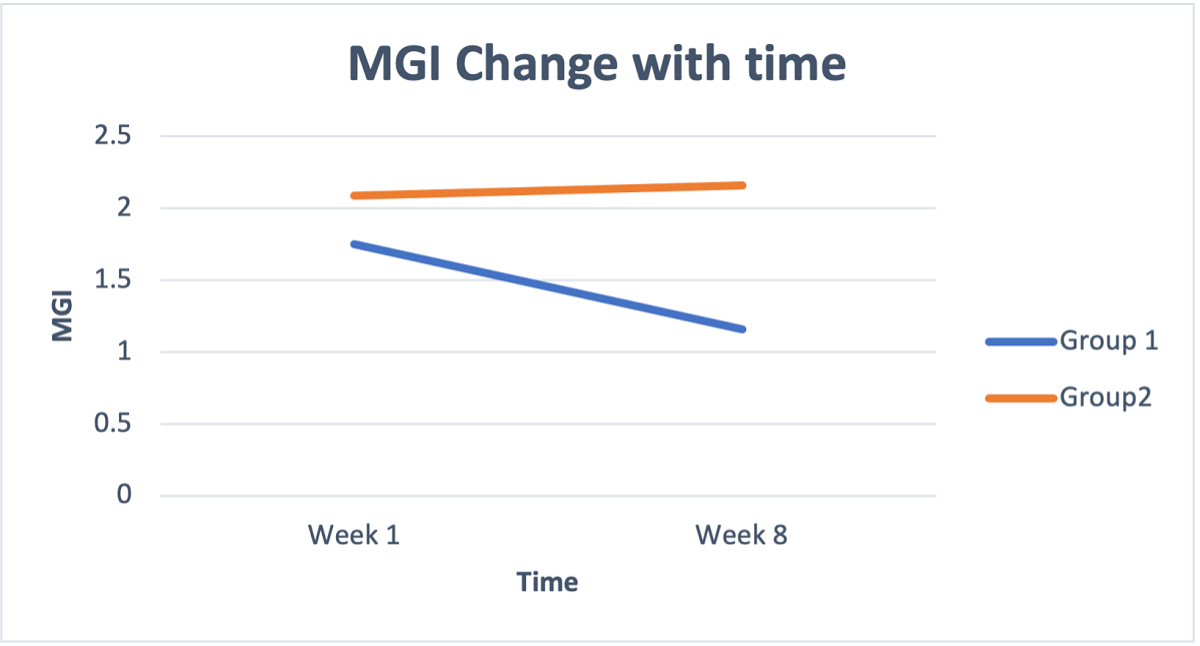
Changes in MGI over the study period. MGI: Modified Gingival Index.

Results of independent *t* tests showed no significant differences between the intervention group (mean 1.75, SD 1.36) and the wait-list control group (mean 2.09, SD 1.55) regarding gingivitis at the start of the study (t_124_= –1.346; *P*=. 18). In contrast, there were significantly lower gingivitis scores in the intervention group (mean 1.16, SD 1.18) compared to the wait-list control group (mean 2.16, SD 1.49; t_91.035_=–3.998; *P*<.001) after 8 weeks. The intervention group showed improvement, whereas there was a decline in gingival health in the wait-list control group although the changes in wait-list control group were not statistically significant (Figure 3).

**Figure 4:**
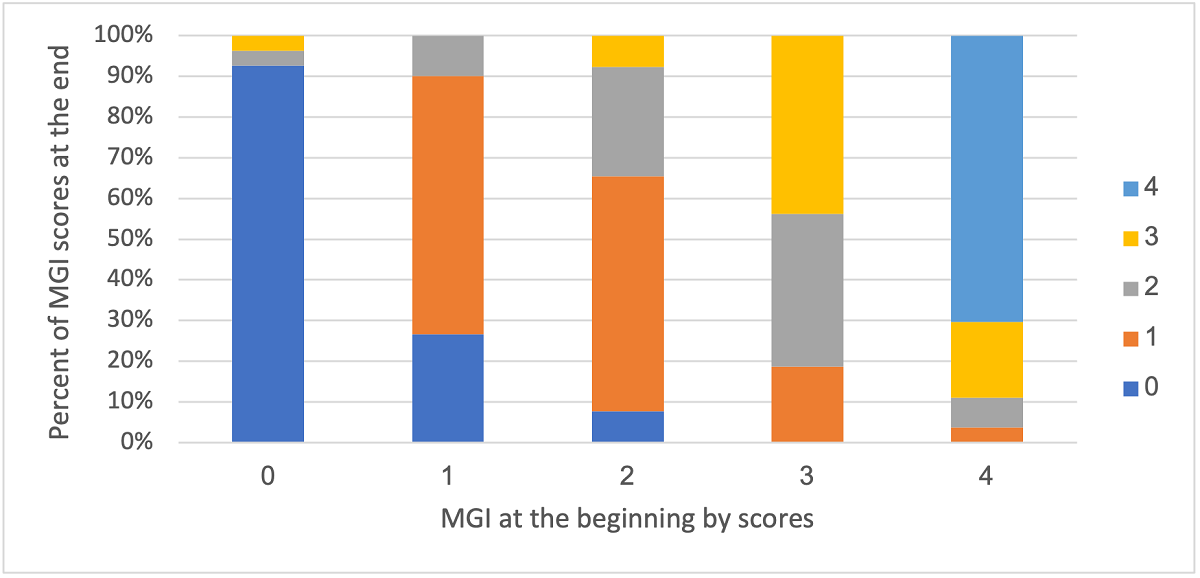
Distribution of MGI scores at the beginning (x-axis) and percentages at the end (y-axis). MGI: Modified Gingival Index.

Repeated measures analysis of the data showed a significant effect in the intervention group (*F*_7,68_=6.672; *P*<.001). A statistically significant difference was found in the dependent variable (MGI) in a linearly negative manner (*F*_1,74_=45.054; *P*<.001); as time passed, the gum condition improved (Figure 3). Of those starting with an MGI=0, 90% (19/21) finished with an MGI=0. Of those starting with an MGI=4, 73% (8/11) finished with an MGI=4 at week 8, and the remaining 27% improved (3/11). Of those starting at an MGI=1, 61% (14/23) finished with an MGI=1, 13% (3/23) with an MGI=2 (ie, worse results), and 26% with an MGI=0 (6/23). Of those starting with an MGI=2, 27% (3/11) remained at an MGI=2 at the end of the study and 64% (7/11) improved (MGI=1: 6/11, 55%; MGI=0: 1/11, 9%). Of those starting with an MGI=3, 44% (4/9) remained at MGI=3, and 56% (5/9) improved (MGI=2: 3/9, 33%; MGI=1: 2/9, 22%; see Figure 4).

**Figure 5:**
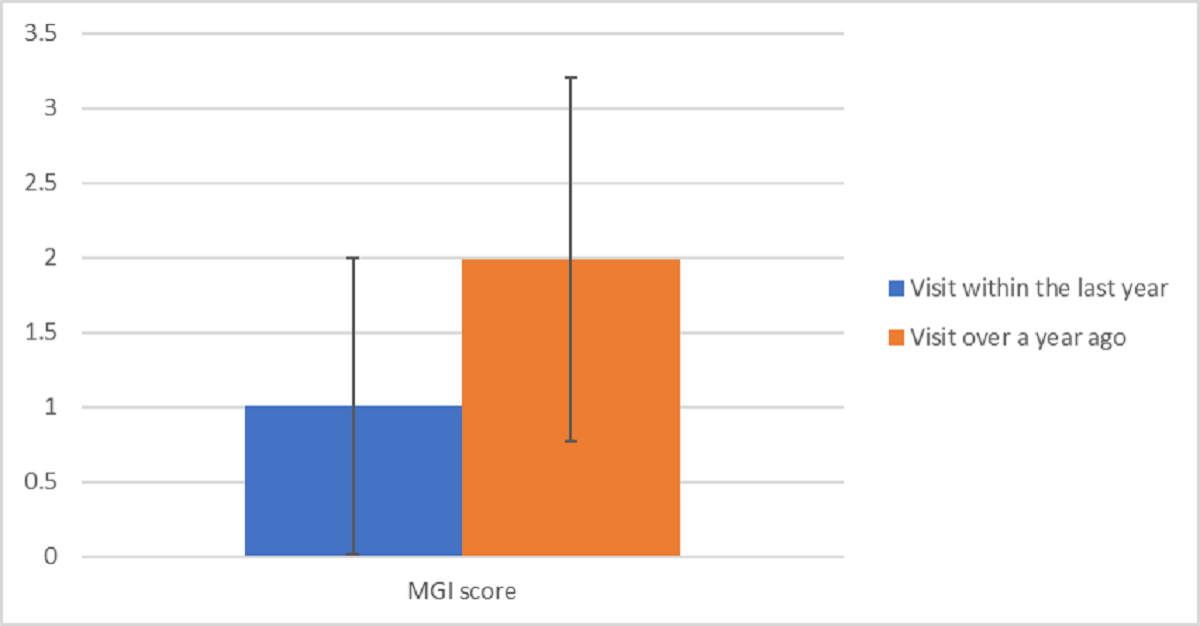
MGI score and timing of last dental visit. MGI: Modified Gingival Index.

Spearman correlation coefficient showed a statistically significant positive association between MGI score and latest visit to the dentist (Spearman correlation coefficient=186; *P*=.04). When the latest visit to the dentist was more recent, the chance of lower MGI was higher. An independent samples *t* test revealed that participants who visited the dentist within the year (mean 0.23, SD 0.76) had significantly less severe gingivitis than did those who had visited more than a year prior (mean 0.46, SD 0.79; t_124_=–1.669; *P*=.049; see Figure 5).

Most participants brushed their teeth twice a day, with 76% (57/75) in the intervention group and 72.5% (37/51) in the wait-list control group answering correctly regarding recommended brushing frequency (intervention group: 66/75, 86.7%; wait-list control group: 46/51, 90.2%). Most reported they rarely used floss (intervention group: 53/75, 70.7%; wait-list control group 43/51, 66.7%) or mouthwash (intervention group: 44/74, 58.7%; wait-list control group 25/51, 49%). The most common value in terms of visits to the dentist in the wait-list control group was “in the last six months” (22/51, 43.1%), whereas in the intervention group, the most common values were “between six months and a year” (24/75, 32%) and “over two years” (24/75, 32%). Most participants knew that the recommended frequency dental visits are “every six months” (intervention group: 43/75, 57; wait-list control group: 39/51, 76.5%). The most common reason for the visit was “treatment” (intervention group: 37/75, 49.3%; wait-list control group: 15/51, 29.4%). In the intervention group, the most common value in terms of a last visit to the dental hygienist was “between one and two years” (23/75, 30.7%), and in the wait-list control group the most common value was “in the last six months” (20/51, 39.2%). Interestingly, most of the participants knew that the recommended frequency of visits to the hygienist is “every six months” (intervention group: 41/75, 54.7%; wait-list control group: 35/51, 68.6%; see Table 2 for details).

**Table 2:** Oral health knowledge and habits.

Question		Intervention control group, n (%) (N=75)		Wait-list control group, n (%) (N=51)
**How often do you brush your teeth?**				
	Once a day	17 (22.7)	12 (23.5)	
	Twice a day	(76) 57	37 (72.5)	
	More than twice a day	0 (0)	0 (0)	
	Rarely/irregularly	1 (1.3)	2 (3.9)	
**As far as you know, what is the recommended frequency for daily brushing?**				
	1	8 (10.7)	4 (7.8)	
	2	65 (86.7)	46 (90.2)	
	More than 2	1 (1.3)	0 (0)	
	Do not know	1 (1.3)	1 (2)	
**How often do you floss?**				
	Once a day	9 (12)	7 (13.7)	
	Twice a day	3 (4)	0 (0)	
	Two to three times a week	6 (8)	4 (7.8)	
	Once a week	1 (1.3)	4 (7.8)	
	Two to three times a month	0 (0)	1 (2)	
	Rarely/irregularly	53 (70.7)	34 (66.7)	
	Never	3 (4)	1 (2)	
**How often do you use mouthwash?**				
	Once a day	7 (9.3)	5 (9.8)	
	Two to three times a week	2 (2.7)	3 (5.9)	
	Once a week	1 (1.3)	3 (5.9)	
	Two to three times a month	4 (5.3)	4 (7.8)	
	Rarely/irregularly	44 (58.7)	25 (49.0)	
	Never	17 (22.7)	11 (21.6)	
**When was the last time you visited the dentist?**				
	In the last six months	19 (25.3)	22 (43.1)	
	Between six months and a year	24 (32)	12 (23.5)	
	Between one and two years	7 (9.3)	10 (19.6)	
	Over two years	24 (32)	6 (11.8)	
	Never	1 (1.3)	1 (2)	
**What was the reason for visiting the dental clinic?**				
	Routine dental examination	11(14.6)	10 (19.6)	
	Treatment by a hygienist	11(14.6)	10 (19.6)	
	Treatment such as restoration, tooth extraction, and root canal treatment	37 (49.3)	15 (29.4)	
	Treatment such as bridge, dentures, orthodontics, and dental implants	12 (16)	13 (25.5)	
	Gingival treatment	2 (2.7)	3 (5.9)	
	Treatment after an accident or fall	2 (2.7)	0 (0)	
**What is the recommended frequency of dental visits?**				
	Every six months	43 (57.3)	39 (76.5)	
	Every year	23 (30.7)	10 (19.6)	
	Every two years	4 (5.3)	0 (0)	
	Do not know	5 (6.7)	2 (3.9)	
**When was the last time you visited a** **hygienist?**				
	In the last six months	17 (22.7)	20 (39.2)	
	Between six months and a year	17 (22.7)	9 (17.6)	
	Between one and two years	23 (30.7)	8 (15.7)	
	Over two years	12 (16)	13 (25.5)	
	I have never visited	6 (8)	1 (2)	
**What is the recommended frequency of visiting the hygienist?**				
	Every six months	41 (54.7)	35 (68.6)	
	Every year	22 (29.3)	12 (23.5)	
	Every two years	5 (6.7)	2 (3.9)	
	Do not know	7 (9.3)	2 (3.9)	

## Discussion

This novel observational study used an mHealth app (iGAM) to examine whether dental selfies reduce gingivitis as determined by the MGI score and investigated the interval between photograph submissions needed to enable an improvement.

COVID-19 has spread rapidly across the world since December 2019, and, in order to reduce infection, social distancing has been recommended [19,20]. Since people rarely go to the dentist if they are asymptomatic [21], we assumed that gingivitis will be neglected and worsen, highlighting a need for remote monitoring in dentistry. mHealth apps are technological tools that can remotely maintain patient care. Indeed, during the COVID-19 period, the number of mHealth apps available and the number of users have increased [22-24].

This observational study demonstrated that a dental mHealth app iGAM can reduce gingivitis. At the beginning of the study, all participants filled in a questionnaire regarding oral hygiene habits and knowledge of oral health maintenance recommendations. Most of the participants were in their third decade of life, men, secular, single, employees and students, healthy, and nonsmokers. These characteristics are consistent with other studies on the use of mobile medical apps [25-28].

We found that the participants with more recent dental visits had significantly better gum health and found that weekly use of the app not only raised awareness of gum health but actually led to its improvement. Studies have shown that the more medical awareness a person has, the greater tendency to use mHealth apps [29-31].

Those taking weekly pictures had reduced gingivitis scores, whereas the other group showed no improvements, and their gum condition slightly deteriorated (the decline in the second group was not statistically significant). Similarly, other observational studies using mHealth apps in which the researcher does not provide any feedback have shown that the apps improve health (eg, reducing asthma attacks) [32-34].

We found a significantly positive correlation between the gum condition at the beginning of the study and at the end of the study in participants with excellent (MGI=0) or terrible (MGI=4) gums. For the remaining participants with scores not at the limits of the index, gingival health improved (ie, MGI scores were lower). There are several possible reasons for these interesting and unique findings.

First, regarding the limits of the MGI, we assume that for an individual with optimal oral health (MGI=0) who knows how to maintain their mouth, no improvement is possible, and therefore they do not need to use the app. Conversely, an individual with the worst score (MGI=4) is probably aware of their poor condition because they have bleeding, swollen gums, and frequent bad breath, and have done nothing about it, so an app is unlikely to change their behavior.

Second, participants with MGI scores between 1 and 3 demonstrated a statistically significant improvement in gum health. Studies have found that most of the population has scores in this range [35-37], with the occasional appearance of signs of inflammation. Therefore, the app assisted in promoting and maintaining gum health over time.

In the examination of knowledge and oral hygiene habits, no association was found between knowledge and behavior. Furthermore, although most participants were familiar with the accepted guidelines for maintaining oral health, they only performed some of them. Most of the participants said they brush twice daily, but rarely use brushing aids. Furthermore, the main reason for visiting the dentist was pain, even though most participants knew that the recommendation is to go to the dentist twice a year for an examination.

More participants in the wait-list control group failed to complete the requirements of the study, even though they had less to do, as they took 2 photographs compared to 8. It seems that having to perform a task every 8 weeks was more challenging than doing so each week. It cannot be argued that participants forgot the date of the photo, as the app sent reminders to each participant on the day they needed to take the photograph. Studies have found that apps with regular activities are more likely to be used [38].

Our study was limited in 7 main aspects. (1) A causal relationship cannot be established from an observational study; however, many investigations on mHealth apps use this methodology, and predict clinical values from their outcomes. (2) We only enrolled participants fluent in Hebrew, which might have increased the risk for selection bias. The app should be translated into the other official languages of Israel (ie, English and Arabic ) in order to gather data and make more generalizable findings. (3) Only Android users were enrolled, which might have increased the risk for socioeconomic bias. For example, a survey [39] among 139,000 US smartphone users showed that Android users have less education than do iPhone users. (4) The advertisement for the study clearly invited participants aged 18 years and above, yet the mean age of the study participant was ~27; this possible selection bias might have been due to the advertisements being posted in academic institutions and university hospitals. A larger-scale study with participants from different age groups would yield more generalizable findings. (5) The 8-week time period for self-monitoring was selected for 2 reasons: gingivitis heals within 10 to 14 days with proper oral hygiene, so improvement is more immediately evident; and most studies on gingivitis are for this period, and thus a longer study might have showed more significant differences between the groups. (6) The questionnaire used was developed exclusively for this study, which limits comparison with questionnaires with established validity. (7) The high dropout rates, especially in the wait-list control group, should not be taken for granted. The impact of the high dropout rate, particularly of the wait-list control group, on the results needs further investigation using qualitative research methods to understand the acceptance and usability of the iGAM mHealth app among the dropouts and those completing the study. We will publish this data in the third paper in this series.

This observational study found that a dental selfie taken once a week using an mHealth app (iGAM) reduces the signs of gingivitis and promotes oral health. In this period of the COVID-19 pandemic where social distancing recommendations may be causing people to avoid dental clinics, this app can remotely promote gum health.

## Abbreviations

mHealth: mobile health
MGI: Modified Gingival Index
